# Community assembly in *Nothobranchius* annual fishes: Nested patterns, environmental niche and biogeographic history

**DOI:** 10.1002/ece3.2851

**Published:** 2017-03-08

**Authors:** Martin Reichard, Michal Janáč, Matej Polačik, Radim Blažek, Milan Vrtílek

**Affiliations:** ^1^ Institute of Vertebrate BiologyAcademy of Sciences of the Czech RepublicBrnoCzech Republic

**Keywords:** Africa, altitudinal gradient, checkerboard pattern, dispersal, ephemeral pools, temporary water body

## Abstract

The assembly of local communities from regional species pools is shaped by historical aspects of distribution, environmental conditions, and biotic interactions. We studied local community assembly patterns in African annual killifishes of the genus *Nothobranchius* (Cyprinodontiformes), investigating data from 168 communities across the entire range of regionally co‐existing species. *Nothobranchius* are small fishes associated with annually desiccating pools. We detected a nested pattern of local communities in one region (Southern Mozambique, with *Nothobranchius furzeri* as the core and dominant species), but no nestedness was found in the second region (Central Mozambique, with *Nothobranchius orthonotus* being the dominant species). A checkerboard pattern of local *Nothobranchius* community assembly was demonstrated in both regions. Multivariate environmental niche modeling revealed moderate differences in environmental niche occupancy between three monophyletic clades that largely co‐occurred geographically and greater differences between strictly allopatric species within the clades. Most variation among species was observed along an altitudinal gradient; *N. furzeri* and *Nothobranchius kadleci* were absent from coastal plains, *Nothobranchius pienaari*,* Nothobranchius rachovii*, and *Nothobranchius krysanovi* were associated with lower altitude and *N. orthonotus* was intermediate and geographically most widespread species. We discuss implications for ecological and evolutionary research in this taxon.

## 1 Introduction

The principles of biological community assembly are of central importance to ecologists (Connor, Collins, & Simberloff, 2013; Connor & Simberloff, 1979; Diamond, 1975; Gaston et al., 2000; Götzenberger et al., 2012; Henriques‐Silva, Lindo, & Peres‐Neto, 2013; Holyoak, Leibold, & Holt, 2005; Hubbell, 2001; Parr, 2008; Ulrich & Gotelli, 2007). The species occurrence is contingent upon its evolutionary and biogeographic history (historical aspects of distribution), environmental characteristics (habitat suitability) and the presence of other species (biotic interactions). The composition of local communities is restricted by diversity of the regional species pool, setting the upper limit to their alpha diversity, and is shaped by a set of “assembly rules” related to the ecological niche and dispersal abilities of potentially co‐occurring species (i.e., meta‐community) (Diamond, 1975; Gaston et al., 2000; Henriques‐Silva et al., 2013; Hubbell, 2001). The assembly patterns identify the non‐random component in local community composition by comparing certain parameters of an observed dataset with the same parameters in multiple randomized datasets (Connor & Simberloff, 1979; Götzenberger et al., 2012).

Nestedness and negative species co‐occurrence are two main patterns of local community assembly. The nested pattern describes the positive co‐occurrence of certain species, with species‐poor communities being predictable subsets of species‐richer communities. Negative species co‐occurrence tends to produce checkerboard patterns where some species combinations are absent, while the distribution of other species can still be positively associated (Diamond, 1975). Historical, biotic and abiotic factors can all drive nested and checkerboard patterns (Connor et al., 2013; Ulrich & Gotelli, 2007). To understand which processes underpin the observed distributional patterns, examination of biotic and abiotic species–occurrence associations is necessary.

The concept of ecological niche, describing species' response to the distributions of resources and potentially co‐existing taxa, serves as a basis for assessing the ecological similarities and differences among species (Barve et al., 2011). The ecological niche characterizes the conditions for species to persist and the degree of niche overlap between potentially co‐existing species determines the pattern of their local co‐occurrence, including environmental aspects of the ecological niche. However, checkerboard patterns can arise for reasons other than inter‐specific competition. Difference in habitat preferences, recent geographic speciation, and constraints on dispersal can all lead to negative co‐occurrence between species (Connor et al., 2013).

Meta‐communities (sets of local communities linked by potential dispersal) and their local subsets vary widely in species richness. Species‐rich communities contributed most to our current understanding of the assembly of local communities (Holyoak et al., 2005). Species‐poor communities in well‐defined habitat patches are more amenable to accurate census. However, very low species diversity leads to low statistical power of some standard analytical procedures that have been developed for species‐richer communities (Barve et al., 2011; Götzenberger et al., 2012; Henriques‐Silva et al., 2013). For taxonomically defined, species‐poor communities, the assembly rules can be easily dissected to specific combinations of species and directly related to particular environmental factors.

We studied local community assembly patterns in a clade of African annual fishes. Annual killifish of the genus *Nothobranchius* (Cyprinodontiformes, Nothobranchiidae) are small fishes that are tightly associated with ephemeral pools in the East African savannah (Wildekamp, 2004). The pools only exist for a short period during and after the rainy season, and annual killifishes are the only teleost fishes adapted to these temporary habitats. Every year, fish hatch from desiccation‐resistant eggs when the pools fill with rainwater (Polačik, Donner, & Reichard, 2011) and complete their life cycle during 1–11 months (Blažek, Polačik, & Reichard, 2013; Reichard, 2015), with eggs surviving the dry period buried in the sediment (Cellerino, Valenzano, & Reichard, 2016; Reichard, Cellerino, & Valenzano, 2015). Several *Nothobranchius* species often inhabit the same pool (Reichard, 2015; Reichard, Polačik, & Sedláček, 2009; Watters, 2009; Wildekamp, 2004). When *Nothobranchius* species co‐exist in a pool, limited space does not permit any substantial microhabitat separation. The diet of co‐occurring species is largely overlapping (Polačik, Harrod, Blažek, & Reichard, 2014; Polačik & Reichard, 2010); there is no apparent trophic specialization. Other teleost fishes only invade temporary pools in the event of substantial flooding, except those in the flat floodplains of the lower reaches of major rivers, where co‐existence with non‐annual fishes is more frequent. In such cases, *Nothobranchius* fishes are constrained to particular microhabitats within a floodplain wetland matrix, not being able to sustain stable populations in direct competition with other teleost fishes (Reichard, 2015). Other vertebrates in temporary pools are *Protopterus* spp. lungfishes and tadpoles (Reichard, Polačik, Blažek, & Vrtílek, 2014), though their ecological relationship to *Nothobranchius* fishes is not known.

**Figure 1:**
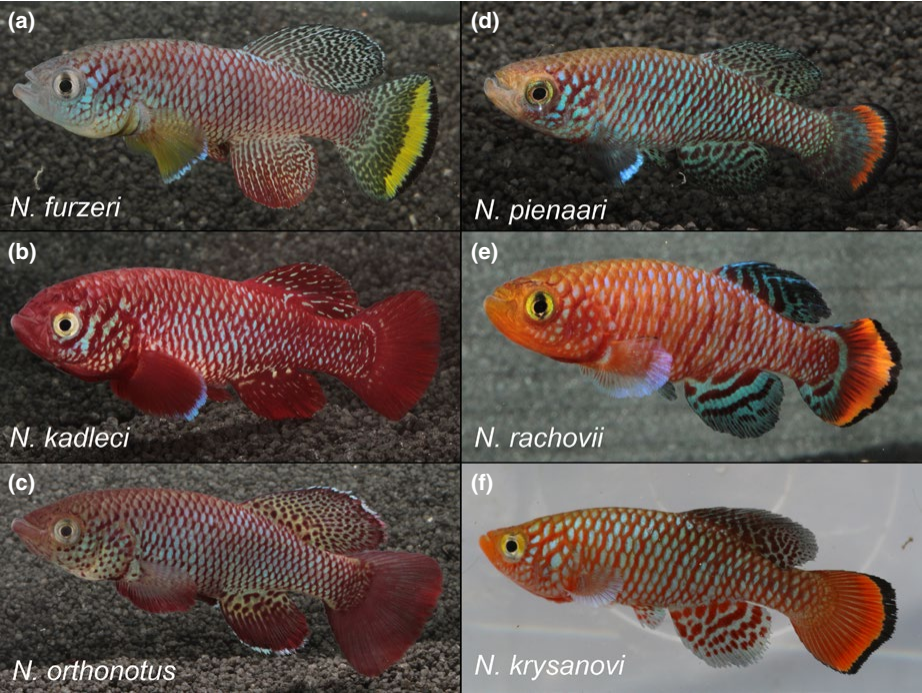
Adult males of all study species

Here, we used an extensive dataset from our long‐term research in southern and central Mozambique. This region (defined by the costal drainages south of the Limpopo and Incomati basins in the south and the extended Zambezi delta in the north) corresponds with the distribution of three well‐separated clades of *Nothobranchius* (Bartáková, Reichard, Blažek, Polačik, & Bryja, 2015; Dorn et al., 2011). The ranges of the three clades overlap (Bartáková et al., 2015). One clade (the O‐clade; Dorn et al., 2011; Bartáková et al., 2015) apparently includes a single, widely distributed species, *Nothobranchius orthonotus* (Peters) (Vrtílek & Reichard, 2016a). The F‐clade (Bartáková et al., 2015; Dorn et al., 2011) consists of two morphologically well‐defined species, *Nothobranchius furzeri* Jubb and *Nothobranchius kadleci* Reichard (Reichard, 2010). The R‐clade (Bartáková et al., 2015; Dorn et al., 2011) contains *Nothobranchius pienaari* Shidlovskiy, Wildekamp, Watters, *Nothobranchius krysanovi* Shidlovskiy, Wildekamp, Watters and *Nothobranchius rachovii* Ahl (Shidlovskiy, Watters, & Wildekamp, 2010). Phylogeographic analysis of all three complexes, based on nuclear and mitochondrial genetic markers, has revealed strong spatial structure of populations, indicative of very low dispersal ability (Bartáková et al., 2013, 2015). All study species are illustrated on Figure 1.

In the present study, we concentrated on understanding the finer‐scale processes that have led to particular local community assembly. Specifically, we asked the following questions. First, is the distribution of the three clades geographically congruent and is the distribution of species within each clade allopatric? Second, are local *Nothobranchius* communities composed of a predictable subset of regionally present species and is the assembly of local communities driven by positive or negative associations among species? Third, is the presence of particular species in local communities explained by local environmental factors?

We used presence/absence data from nine expeditions to the region (between 2008 and 2015) to reconstruct the distribution of each clade and species, and the patterns of their co‐occurrence and nestedness. We then compared the relative abundance of co‐occurring species from a subset of pools and investigated the factors responsible for the observed patterns by analyzing species–habitat associations. We used multivariate environmental niche modeling (Broennimann et al., 2012; Warren, Glor, & Turelli, 2008) to compare niche overlap between the clades and between species within the clades. We then used univariate linear models to identify the key local habitat characteristics important for the presence of particular species.

## 2 Methods

### 2.1 Fish collection

Data were collected during nine expeditions between 2008 and 2015 (8 February–23 March 2008, 6–26 February 2009, 20 February–4 March 2010, 5–16 April 2010, 24 February–13 March 2011, 23 February–16 March 2012, 21 May–7 June 2012, 28 March–16 April 2013, 7–23 March 2015) that targeted various regions of the study area. Most expeditions were completed in the latter part or immediately after the rainy season (February to April), though two expeditions visited the region during May and July. Some sites (especially in remote areas) were sampled only once (55 sites), while some sites were sampled over several years, as part of separate studies (113 sites with *Nothobranchius* presence). Repeated sampling of the same sites usually recovered the same *Nothobranchius* communities within particular pools, though in 20 cases, repeated sampling over years revealed an additional species. All suitable sites were targeted, but we generally omitted pools in close proximity to each other (and likely connected during flooding). Pools directly connected to large rivers were not sampled on the basis of previous knowledge (Wildekamp, 2004) that they do not include *Nothobranchius* fishes. Stagnant pools within the riverbed of smaller temporary streams were included in the sampling design, as they often conform to annual fish habitat characteristics (Reichard, 2015). Over the years, we aimed to sample all accessible regions in Southern and Central Mozambique and the adjacent part of Zimbabwe that were deemed likely to contain *Nothobranchius* fishes.

To collect fish, a 5‐mm‐mesh dip net with a triangular metal frame (45 × 45 cm) on a 1.5‐m wooden pole was used in most sites. Typically, 15–40 hauls were performed at each site but more hauls were performed when fish density was low. Fewer hauls were taken if the site was too small to accommodate 15 hauls and more hauls were sometimes taken when we aimed to collect a large number of individuals for a different purpose (e.g., population genetic analysis). In larger sites, dip net sampling was supplemented by sampling using a seine net (length 2.7 m, depth 0.7 m, mesh size 4 mm), especially when we aimed for a larger sample size. The mesh size used retained adult *Nothobranchius* unselectively and there was no apparent species‐specific bias in the probability of capture. Sampling effort variation within and among years had a negligible effect on species detection. Fish were unambiguously identified to species in the field. Most individuals were returned to the pools, but in some cases a subsample was taken to the laboratory for further research.

All work was carried out in accordance with relevant guidelines and regulations. Sample collection complied with legal regulations of Mozambique (collection licenses: DPPM/053/7.10/08, 175/154/IIP/2009/DARPE, DPPM/083/7.10/10, DPPM/330/7.10/10, DPPM/069/7.10/11, DPPM/088/7.10/12; export licenses: 013/MP/2008, 049MP00518‐A/09, 133/2791/MP/2010, 27/2437, 187MP/2011, 191MP/2012, 238MP/2013 of the Ministry of Fisheries), and general research procedures were approved by the Ethical Committee of the Institute of Vertebrate Biology, in accordance with legal regulations of the Czech Republic. No experimental work was carried out; therefore, no experimental protocols to be approved were available.

### 2.2 Habitat data

We estimated a set of habitat variables for each site. GPS reading (longitude, latitude) was taken during each collection, along with altitude (to the nearest 1 m) using Garmin nüvi 550 (Garmin Ltd.). Over the first 5 years of data collection (2008–2012), a set of habitat variables was taken according to the sampling protocol. The estimates of pool area (estimated to the nearest 10 m^2^), water turbidity (ordinal scale; transparent: bottom visible; turbid: visibility 1–10 cm; very turbid: visibility <1 cm), and substrate structure (ordinal scale; sand; hard clay bottom; mud; soft mud) were included in the data analysis, along with estimated percentage vegetation cover (separately for water lillies [*Nymphaea* spp.], submerged aquatic vegetation and grassy littoral vegetation within the pool, including its margins). Other habitat variables that were collected (e.g., maximum water depth, water temperature and water conductivity) varied considerably both diurnally and seasonally and were not useful in predicting the presence of particular species. Further details on habitat data collection are given in Reichard et al. (2009, 2014).

Given that habitat data were collected over separate years and were subject to intra‐seasonal variability, we averaged continuous and ordinal values across years (pool area, water turbidity, substrate). Pool area had three outliers when analyzed on a (log‐transformed) continuous scale and this resulted in overdispersion of residuals and artificial inflation of confidence intervals. We therefore coded pool size categorically (small: 2.5–100 m^2^, intermediate: 101–10,000 m^2^, large: >10,000 m^2^), which corresponded well to the extent of their inter‐annual variation. Finally, all three vegetation categories varied among years in presence/absence and percentage cover. To provide reliable estimates independent of temporal variation, we scaled them into three categories; absent (A), present but infrequent or variable among years (B), and abundant and always present (C).

### 2.3 Analysis of co‐occurrence

A pairwise co‐occurrence between species with overlapping ranges was quantified using the probabilistic model of species co‐occurrence on presence–absence data (Griffith, Veech, & Marsh, 2015). The analysis compared the observed and expected frequencies of co‐occurrence between the pair of species. The expected frequency is based on a random distribution of each species, independent of the other species. Statistically significant over‐ and under‐representations of particular pairwise associations were interpreted as positive and negative co‐occurrence. We quantified relative species abundance as the relative proportion of individuals of a particular species in a *Nothobranchius* community for a given site, calculating the relative abundance of each species for each combination of species separately. The co‐occurrence analysis was conducted in R 2.14.2 (R Foundation for Statistical Computing, Vienna, Austria), using the *cooccur* library (Griffith et al., 2015). We used Venn diagrams to visualize proportional species co‐occurrence.

### 2.4 Nestedness analysis

Nestedness was analyzed using the number of decreasing fills (NODF) (Almeida‐Neto, Guimarães, Guimarães, Loyola, & Ulrich, 2008) as the nestedness metric. This index can range from 0 (completely random) to 100 (perfectly nested). Statistical significance was tested by comparing the NODF values with 999 random assemblages created according a null model. Choosing the appropriate null model is the most controversial aspect of statistical inference in nestedness analysis (Ulrich, Almeida‐Neto, & Gotelli, 2009). The equiprobable null model (that preserves total number of species occurrences in the original matrix, but does not preserve margin totals) is truly random, but criticized for being prone to type I errors (Ulrich et al., 2009; Wright, Patterson, Mikkelson, Cutler, & Atmar, 1998). Fixed–fixed null models (that preserve both margin totals) are suggested as an alternative, but often fail when matrices are extremely nested, because there are too few possible matrix rearrangements (Almeida‐Neto et al., 2008). This limitation is especially apparent in small‐size matrices, as in our dataset. As a compromise, we chose a null model that constrained only column totals (proportional to species relative abundances; Randnest, or c0) (Jonsson, 2001), and allowed more row rearrangements in our species‐poor matrix. We did not use matrix temperature to estimate nestedness (Atmar & Patterson, 1993), because this method has been criticized for being prone to type I errors (Ulrich et al., 2009; Wright et al., 1998). The nestedness analysis was conducted using the *vegan* library (Oksanen et al., 2012).

### 2.5 Multivariate niche modeling

Niche overlap between clades and between species within clades was measured using the niche similarity test (Warren et al., 2008). This analysis compares realized niche overlap against a series of overlaps calculated from randomized datasets to examine whether similarity between the niches of two taxa is different from that expected by chance (Warren et al., 2008). Using this test, it is also possible to compare environmental niches of species with non‐overlapping geographic ranges (Broennimann et al., 2012). The latter approach, which we have undertaken, measures niche overlap along two‐dimensional space determined by multivariate analysis gradients, weighting densities of species occurrences by densities of environmental factors along these gradients. Analogically, we used the niche equivalency test that examines “niche conservatism in the strictest sense” (Broennimann et al., 2012; Warren et al., 2008). The null hypothesis for this analysis postulates that niches occupied by two entities are identical.

Environmental gradients were constructed using principle component analysis (PCA). All environmental factors (altitude, water turbidity, substrate, littoral, aquatic and *Nymphaea* vegetation) were scaled prior to PCA. Two sites with very high altitude in the range of *N. kadleci*,* N. orthonotus*, and *N. rachovii* were deleted as they represented major outliers (420 and 545 m a.s.l.) and were retrospectively considered to be outside study species distribution. The results of the PCA are given in Table 1. The first two PCA axes explained 31.4% and 17.9% of variation, respectively and were used as environmental gradients (Broennimann et al., 2012). PC1 mainly represented a gradient from turbid, non‐vegetated pools to vegetated and relatively larger pools with clearer water. PC2 mainly represented a substrate gradient (from very soft to relatively hard), with a contribution of water turbidity (Table 1). We used 100 iterations when randomizing the datasets for each niche similarity and niche equivalency test. Niche overlap analyses were conducted using *ecospat* library (Broennimann et al., 2015) in R.

**Table 1:** Percentage of variance explained (Var) and loadings of environmental variables on principle component analysis (PCA) axes

	PCA 1	PCA 2	PCA 3	PCA 4	PCA 5	PCA 6	PCA 7
Var (%)	31.38	17.93	15.26	11.85	9.29	8.17	6.13
Pool size	0.50	−0.05	−0.34	0.77	0.13	−0.09	−0.07
Turbidity	−0.65	−0.55	−0.06	0.02	0.27	0.19	−0.41
Substrate	0.22	0.86	−0.03	−0.13	0.32	0.10	−0.29
Nymphaea	0.74	−0.13	−0.01	−0.06	−0.43	0.44	−0.21
Submersed	0.69	−0.31	−0.07	−0.34	0.03	−0.50	−0.23
Littoral	0.66	−0.32	0.25	−0.11	0.51	0.25	0.23
Altitude	−0.03	−0.04	−0.93	−0.29	0.07	0.12	0.14

### 2.6 Univariate analyses of environmental predictors of species presence

The same set of predictors as in the PCA was used to test the univariate response of particular species presence to each environmental variable. Only sites from within the geographic range of particular species were selected for analysis. Within the R‐clade, three sites without *Nothobranchius* were deleted because it cannot be determined whether they belonged to the range of *N. pienaari* or *N. rachovii*.

Species presence was modeled using a Bernoulli generalized linear model (GLM) with a polynomial effect for altitude (to model constraints at both extremes of the gradient) and linear effects for substrate and turbidity, and pool size, littoral, submersed and *Nymphaea* vegetation that were all converted into three categories. Before applying statistical models a data exploration was undertaken, following the protocol described in Ieno and Zuur (2015). The data were examined for outliers in the response and explanatory variables, homogeneity and zero inflation in the response variable, collinearity between explanatory variables, and the nature of relationships between the response and explanatory variables. We present full models for four study species but backward selection of the minimal adequate model produced concordant results. The number of sites sampled in the *N. rachovii* and *N. krysanovi* ranges was too low to provide reliable estimates from GLM analysis.

## 3 Results

### 3.1 Overview and geographic patterns

**Figure 2:**
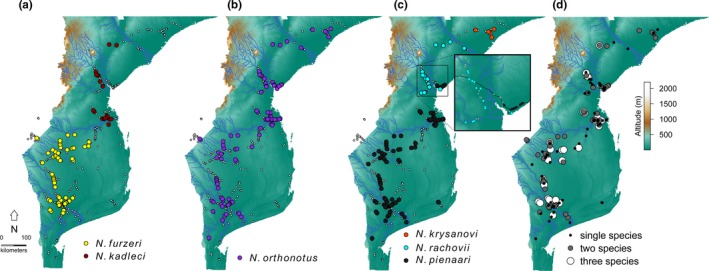
Distribution maps of three study clades and species co‐existence. The presence of (a) *Nothobranchius furzeri* (yellow) an *Nothobranchius kadleci* (dark red), (b) *Nothobranchius orthonotus* (violet), and (c) *Nothobranchius pienaari* (black), *Nothobranchius rachovii* (blue) and *Nothobranchius krysanovi* (red) populations at particular sites. Small empty points represent sampled sites where population of a given clade was absent. (d) A combined dataset illustrating geographic aspect of species co‐occurrence in local *Nothobranchius* communities, where sites with single, two and three co‐existing species are illustrated. An inset details the region with a parapatric distribution of *N. rachovii* and *N. pienaari*. The maps were created in R environment (R Core Team 2015) [packages *sp* (Bivand, Pebesma, & Gomez‐Rubio, 2013), *rgdal* (Bivand, Keitt, & Rowlingson, 2016), *maps* (Becker, Wilks, Brownrigg, Minka, & Deckmyn, 2016) and *GIStools* (Brunsdon & Chen, 2014)]. The altitudinal gradient for Mozambique was downloaded from http://www.diva-gis.org/gdata

Overall, 374 pools were sampled and 168 (45%) contained at least one *Nothobranchius* species. Three species co‐occurred in 35 pools, two species in 62 pools, and 71 pools contained a single species. Within each clade, the ranges of individual species were always allopatric. The ranges of *N. furzeri* and *N. kadleci* (F‐clade) were separated by the River Save, with a single *N. kadleci* population present south of the Save. Within the R‐clade, the ranges of *N. krysanovi* and *N. rachovii* were strictly divided by the River Zambezi. *Nothobranchius rachovii* and *N. pienaari* were parapatric in the region of the Pungwe and Buzi rivers estuaries, though they never co‐occurred in a single pool (Figure 2). Given that *N. furzeri* and *N. kadleci* widely co‐existed with *N. orthonotus* and *N. pienaari* across most of their distribution, we partitioned the study area into *N. furzeri* (i.e., Southern Mozambique) and *N. kadleci* (i.e., Central Mozambique) ranges in the following quantitative analyses.

### 3.2 Patterns of local co‐existence: co‐occurrence, nestedness and relative species abundance

At the level of clades, negative co‐occurrence was found between species from the F‐clade and the R‐clade (*p* = .018). The distribution of clades was significantly nested (NODF = 61.0, *p* = .001).

**Figure 3:**
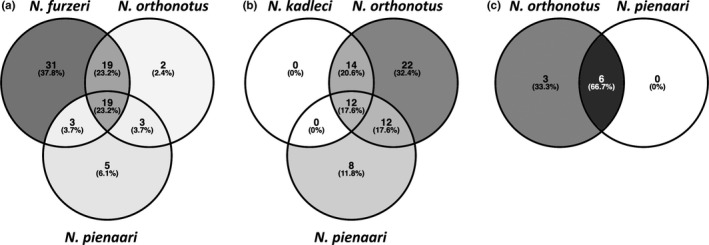
Quantification of species co‐existence in local *Nothobranchius* communities. Venn diagrams visualizing proportional species co‐occurrence (a) within *Nothobranchius furzeri* range, (b) within *Nothobranchius kadleci* range and (c) outside the range of F‐clade. Diagrams were constructed using Venny 2.0.2 (Oliveros, 2007–2015)

**Figure 4:**
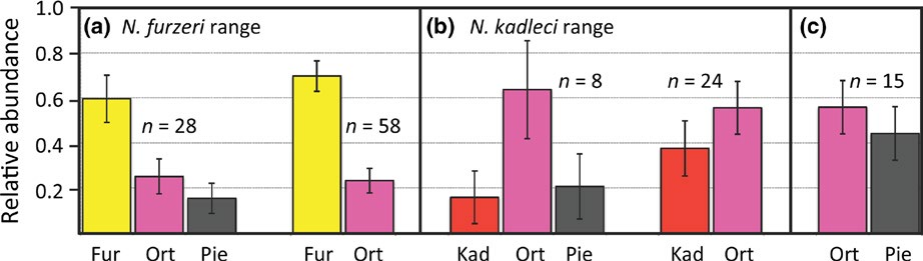
Relative species abundance. Quantitative estimates of relative species abundance were calculated as relative proportion of individuals of a particular species in a *Nothobranchius* community for a given site. Co‐existence between (a) *Nothobranchius furzeri* and its congeners, (b) *Nothobranchius kadleci* and congeners, and (c) *Nothobranchius orthonotus* and *Nothobranchius pienaari* are illustrated. Mean values with 95% confidence intervals were calculated from a set of all separate species ratios. The number of communities used for quantitative estimates is given for each combination

In Southern Mozambique, *N. furzeri* was the core species. Positive co‐occurrence between *N. orthonotus* and *N. pienaari* (*p* = .004) and negative co‐occurrence between *N. furzeri* and *N. pienaari* (*p* = .004) was demonstrated. However, all three species also co‐occurred relatively frequently in three‐species communities (Figure 3a). Their distribution was significantly nested (NODF = 64.7, *p* = .001). Quantitatively, *N. furzeri* was the dominant species, followed by *N. orthonotus* and *N. pienaari* when all three species co‐existed in a single pool. *Nothobranchius furzeri* populations were also numerically larger than populations of the other co‐existing species in two‐species communities with *N. orthonotus* (Figure 4a). Exclusive co‐existence of *N. furzeri* and *N. pienaari* was too rare (two pools) to be analyzed quantitatively.

In Central Mozambique, *N. orthonotus* was the most frequent species and *N. kadleci* and *N. pienaari* (replaced by *N. rachovii* in the northern part of *N. kadleci* range) shared a secondary species position (Figure 3b). *Nothobranchius orthonotus* and *N. kadleci* co‐occurred more often than expected by chance (*p* = .012), while the presence of *N. orthonotus* and R‐clades species (*N. pienaari* and *N. rachovii*) was negatively associated (*p* = .001). No significant nestedness in the local community assembly was detected (NODF = 61.2, *p* = .107). *Nothobranchius orthonotus* was also the numerically dominant species and, at sites where all three species co‐existed, *N. pienaari* and *N. kadleci* occurred at similar abundances. In two‐species communities with either *N. kadleci* or *N. pienaari*, quantitative dominance of *N. orthonotus* was relatively smaller than in three‐species communities (Figure 4b).

### 3.3 Ecological niche modeling

**Figure 5:**
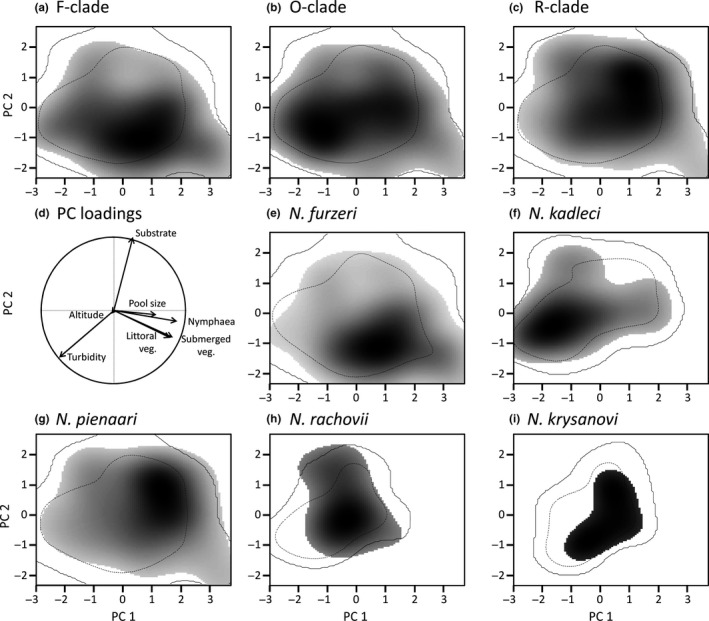
Niche similarity among and within clades. Two‐dimensional visualization of niches along environmental gradient detected by the principle component analysis (PCA). (a–c) clade‐specific niches with (d) correlation of environmental predictors with PCA axes, and species‐specific niches for (e) *Nothobranchius furzeri*, (f) *Nothobranchius kadleci*, (g) *Nothobranchius pienaari*, (h) *Nothobranchius rachovii* and (i) *Nothobranchius krysanovi*. Gray shading indicates density of occurrence along two PCA gradients. The solid and dashed contours indicate 100% and 50% of available environment, respectively

Using a subset of sites for which detailed habitat characteristics were available (257 pools; 124 with a *Nothobranchius* species present), we calculated niche similarity and niche equivalency to measure environmental niche overlaps. The PCA yielded the first axis (PC1) representing a gradient from turbid, non‐vegetated pools to vegetated and relatively larger pools with clearer water. The second axis (PC2) mainly represented a substrate gradient (from very soft to relatively hard), with a contribution of water turbidity (Table 1, Figure 5d).

At the level of clades, the niche equivalency and niche similarity tests detected a difference in environmental niche between the F‐ and R‐clades. The O‐clade niche was similar to both the R‐ and F‐clade niches using either test (Table 2a) and was placed in an intermediate position (Figure 5a–c). Within the F‐clade, the environmental niches of *N. furzeri* and *N. kadleci* were clearly differentiated (Table 2b, Figure 5e–f). Within the R‐clade, the environmental niches of *N. pienaari* and *N. rachovii* were also largely different, though no difference was indicated by one of niche similarity tests (Table 1c). There were too few *N. krysanovi* populations to permit analysis for this species, despite a superficial similarity between the *N. rachovii* and *N. krysanovi* niches (Figure 5g–i).

**Table 2:** Observed niche overlaps and their statistical significance. The number of pools used to construct the niche for each taxon (*N*) and values of Schoener's *D* between environmental niches occupied by *Nothobranchius* clades and species within the clades (*D*)

	*N*	*D*	Niche similarity (1–>2)		Niche similarity (2–>1)		Niche equivalency test	
			*p*	Interpretation	*p*	Interpretation	*p*	Interpretation
Among clades								
R‐clade versus O‐clade	257; 257	0.747	.040	Similar	.010	Similar	.060	Identical
R‐clade versus F‐clade	257; 234	0.660	**.100**	**Not similar**	**.060**	**Not similar**	**.050**	**Not identical**
O‐clade versus F‐clade	257; 234	0.829	.010	Similar	.010	Similar	.090	Identical
Within F‐clade								
*Nothobranchius furzeri* versus *Nothobranchius kadleci*	156; 78	0.401	**.119**	**Not similar**	**.594**	**Not similar**	**.020**	**Not identical**
Within R‐clade								
*Nothobranchius pienaari* versus *Nothobranchius rachovii*	203; 34	0.309	.020	Similar	**.307**	**Not similar**	**.020**	**Not identical**

*p*‐Values represent statistical significance of the niche overlap tests, followed by their interpretation according to Warren et al. (2008). Note contrasting interpretation of statistical significances for niche similarity and niche equivalency tests. Interpretations implying significant niche separation (and their *p*‐values) are highlighted in bold.

### 3.4 Environmental predictors of species presence

#### 3.4.1 *Nothobranchius furzeri*

**Figure 6:**
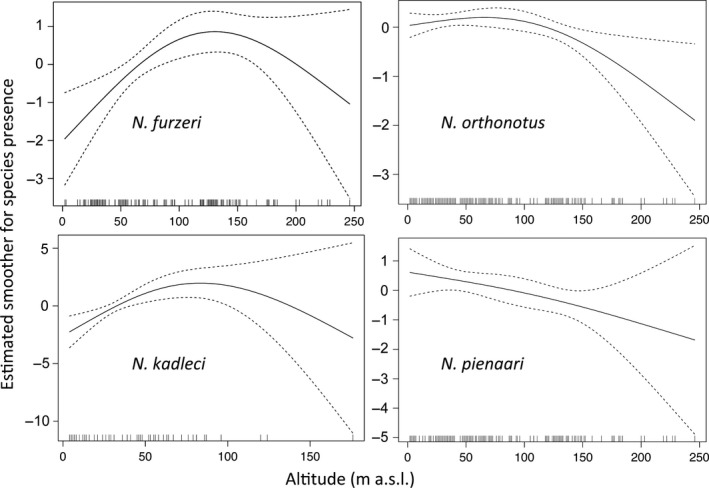
Univariate responses of species presence to altitudinal gradient. The response curves were constructed using general additive models, with three knots fixed to account for second‐order polynomial fit. Mean smoother estimates visualize the probability of particular species presence along altitudinal gradients, with 95% confidence intervals

Detailed habitat data were available from 154 pools within the potential range of this species, with 68 *N. furzeri* populations found (56% of the pools). The presence of *N. furzeri* was most significantly related to altitude (Table 3), with populations absent from coastal areas (no occurrence below 24 m a.s.l.) and rarely recorded at high altitude (Figure 6). *Nothobranchius furzeri* was more common at sites with a soft substrate and positively associated with littoral, submerged and *Nymphaea* vegetation (Table 3).

**Table 3:** ANOVA results of linear models with binomial error structure predicting presence of *Nothobranchius* spp. populations as a function of environmental characteristics

	*df*	(a) *Nothobranchius furzeri* (*n* = 152)		(b) *Nothobranchius kadleci* (*n* = 76)		(c) *Nothobranchius orthonotus* (*n* = 256)		(d) *Nothobranchius pienaari* (*n* = 200)	
		χ^2^	*p*	χ^2^	*p*	χ^2^	*p*	χ^2^	*p*
Altitude	1	0.84	.359	**6.85**	**.009**	**7.17**	**.007**	**10.17**	**.001**
Altitude (^2)	1	**5.35**	**.021**	**12.75**	**<.001**	**5.31**	**.021**	0.00	.969
Substrate	1	**4.81**	**.028**	0.11	.739	2.94	.087	3.17	**.075**
Turbidity	1	0.23	.633	**3.87**	**.049**	0.25	.614	2.99	.084
Pool size	2	**9.33**	**.009**	1.91	.385	0.32	.853	1.18	.555
Littoral veg.	2	**32.82**	**<.001**	0.52	.772	**15.28**	**<.001**	**8.74**	**.013**
Submersed v.	2	5.67	.059	2.12	.145	0.15	.930	2.42	.298
*Nymphaea* v.	2	**9.25**	**.010**	1.15	.563	3.18	.204	**6.29**	**.043**

Statistically significant results are highlighted in bold. Altitudinal effect is visualized in Figure 6. All significant relationships between *Nothobranchius* population presence and vegetation are positive.

#### 3.4.2 Nothobranchius kadleci

Twenty‐one *N. kadleci* populations were found across 78 pools investigated within the potential range of this species (27%). As with *N. furzeri*, the *N. kadleci* was absent from coastal areas (a single population present at 16 m a.s.l., others at 26 m a.s.l. and higher) and high‐altitude sites (Figure 6). *Nothobranchius kadleci* were also more likely to occur at sites with higher water turbidity (Table 3).

#### 3.4.3 Nothobranchius orthonotus

*Nothobranchius orthonotus* populations were found in 93 of 258 pools sampled within its range (36%). This species was present in coastal plains (lowest recorded altitude 2 m a.s.l.) and the probability of its presence declined above altitudes of approximately 100 m a.s.l. (Figure 6). This species was positively associated with pools that contained more littoral vegetation (Table 3).

#### 3.4.4 Nothobranchius pienaari

The species was found in 45 of 202 pools sampled within its range (22%). There was a negative linear relationship between *N. pienaari* presence and altitude (Figure 6), though *N. pienaari* was found as high as 181 m a.s.l. *N. pienaari* populations were positively associated with sites that contained littoral and *Nymphaea* vegetation, and tended to have more solid substrate (Table 2).

## 4 Discussion

Interactions between local and regional processes are central in shaping local community assembly (Barve et al., 2011; Connor & Simberloff, 1979; Connor et al., 2013; Diamond, 1975; Gaston et al., 2000; Götzenberger et al., 2012; Henriques‐Silva et al., 2013; Hubbell, 2001; Ulrich & Gotelli, 2007). The diversity of the regional species pool sets the upper limit to potential alpha diversity and the ability of species to disperse within the region enables local communities to diverge. Different local environmental conditions and species interactions then shape the particular composition of local communities. Previous research has documented a strong role of historical vicariance on large‐scale distributional patterns of *Nothobranchius* fishes. The genus is separated into four major allopatric lineages (Dorn, Musilová, Platzer, Reichwald, & Cellerino, 2014), of which the Southern lineage, consisting a total of six species assigned to three monophyletic clades, was investigated in the present study throughout its entire distribution. Phylogeographic analysis of all three complexes, based on nuclear and mitochondrial genetic markers, has revealed strong spatial structure of populations, indicative of very low dispersal ability (Bartáková et al., 2013, 2015).

In the present study, an altitudinal gradient was demonstrated to play a role in geographic distribution of the main clades (and, consequently, of species within the clades). The F‐clade, with *N. furzeri* and *N. kadleci*, was absent from coastal plains, while the R‐clade (*N. pienaari*,* N. rachovii*,* N. krysanovi*) was underrepresented (though not absent) at relatively higher altitude. This pattern may be related to historical dispersal or environmental conditions. We think that a major role for historical dispersal (or a lack of it) is unlikely. While the source, routes, and timing of *Nothobranchius* colonization of the study area are currently not known, it is very likely that F‐clade species would readily disperse further downstream along major river basins within their range. Significant downstream dispersal along some streams has been demonstrated in *N. furzeri*, with genetic similarity (in terms of non‐significant *F*_ST_ values across multiple microsatellite loci) between relatively distant sites (75 km, with a difference of 50 m a.s.l.) along one of the temporary streams (Bartáková et al., 2013). Consequently, large‐scale flooding must certainly disperse *N. furzeri* and *N. kadleci* individuals into low‐altitude sites.

We suggest a major role for local conditions on the lack of F‐clade species in coastal plains. Importantly, the altitudinal gradient in the study area coincides with a gradient of aridity where high‐altitude sites are significantly drier, with lower total rainfall, higher evapotranspiration rates, and lower predictability of rains (Terzibasi Tozzini et al., 2013; Vrtílek & Reichard, 2016b). The coastal plains are more humid, with temporary pools inundated for a longer period (Blažek et al., 2017; Terzibasi Tozzini et al., 2013). The flat topography of the coastal region likely connects isolated habitats with permanent water bodies more frequently than pools at a higher altitude. Hence, coastal plain sites are more likely to be invaded by non‐annual fishes (Reichard, 2015) and *N. furzeri* and *N. kadleci* may be more sensitive to the presence of non‐annual fishes than the other *Nothobranchius* species. The exclusion of F‐clade species from coastal plains cannot be directly associated with increased salinity; water conductivity in most coastal plain pools is within the range recorded from higher‐altitude sites (Reichard et al., 2009). Another reason for the absence of *N. furzeri* and *N. kadleci* in coastal regions could be that their eggs and embryos may have more stringent substrate requirements to successfully complete development. Inappropriate conditions in the coastal region unlikely include a shorter duration of the dry period for embryo development, because *N. furzeri* and *N. kadleci* embryos can complete their development in less than a month (Blažek et al., 2013; Polačik, Blažek, & Reichard, 2016). Nevertheless, the more humid coastal region receives more seasonal and non‐seasonal rainfall (Blažek et al., 2017; Terzibasi Tozzini et al., 2013). It is possible that the eggs of *N. furzeri* and *N. kadleci* are more prone to non‐seasonal rains, triggering their hatching outside the appropriate season and compromising population persistence. This possibility is amenable to experimental testing.

The upper limit of distribution of *Nothobranchius* species is constrained by altitude; the presence of all species was rare above 150 m a.s.l. This lack of *Nothobranchius* populations is apparently related to regional topography. Despite the absence of *Nothobranchius* from active alluvial plains, they are still restricted to relatively flat depositional areas with particular substrate conditions. Higher‐altitude sites in the study area are mainly in regions with a naturally steeper gradient where the conditions producing seasonal pools are rare. However, populations of three study species are known from sites at an altitude above 320 m a.s.l. The type locality of *N. furzeri* is Sazale Pan in Gona Re Zhou National Park (GRZ‐NP) in Zimbabwe (Jubb, 1971) (located 10 km from Mozambican border), at an altitude of approximately 350 m a.s.l., though we were unable to locate this population during the expedition in 2015. Extensive sampling within GRZ‐NP and the surrounding region revealed only two sites where *Nothobranchius* fishes were present. *Nothobranchius furzeri* and *N. orthonotus* populations co‐existed at altitudes of 325 and 340 m a.s.l., respectively, located 2 and 6.5 km from the Mozambican border. Additionally, *N. orthonotus* and *N. pienaari* were reported co‐existing in two pools (Pumbe Picket and N'tomeni Spruit) in the Kruger National Park, Republic of South Africa, at an altitude of almost 400 m a.s.l. (Watters, Wildekamp, & Cooper, 2009) Given extensive research effort in the area, it is likely that these represent isolated populations (Shidlovskiy et al., 2010), located at the border with Mozambique. It remains to be investigated whether high‐altitude sites represent ancient populations remaining from a period of more suitable environmental conditions (i.e., in regions currently too dry to sustain more viable populations that were frequent in that region in the past), ancient populations that acted as sources to colonize lower‐altitude sites, or recent colonization events.

We demonstrated that local *Nothobranchius* communities showed the most frequent species are also the most numerous. This positive abundance–occupancy relationship is frequently reported from other communities (Gaston, 2003). Further, the presence of dominant species in a community (*N. furzeri* and *N. orthonotus* in the Southern and Central Mozambique, respectively) was negatively associated with presence of the R‐clade species (*N. pienaari* and *N. rachovii*). A checkerboard pattern was also identified; in two‐species communities, *N. kadleci* and *N. pienaari* (or *N. rachovii*, respectively, in the northern part of the *N. kadleci* range) never co‐existed. Similarly, *N. furzeri* co‐existed with *N. pienaari* in a two‐species community in only two sites (3% of 76 sites with a population of *N. furzeri*). One of the sites (MZCS 119) was visited repeatedly and the qualitative community composition was stable across years. *Nothobranchius orthonotus* absence at this site is, therefore, unlikely related to an inadequate sampling. It is interesting that the species negatively associated in two‐species communities were frequently co‐existing in three‐species communities. We suggest that the observed checkerboard pattern is related to environmental characteristics rather than biotic interactions between *Nothobranchius* species. F‐clade and R‐clade species were associated with most dissimilar positions on environmental niche gradients, especially along the vegetation–turbidity axis, and their environmental niches overlap in the segment of *N. orthonotus* preference (Figure 5). The range of environmental characteristics suitable for co‐existence of F‐clade and R‐clade species was, therefore, always suitable also for *N. orthonotus*. The absence of *N. orthonotus* is some of these pools was likely a result of stochastic local extinctions (or a lack of colonization); species from all three clades co‐existed frequently where habitat conditions were favorable for all three species (Figure 3a,b). Hence, the rarity of local communities with exclusive co‐existence of F‐clade and R‐clade species was apparently mediated by environmental conditions rather than inter‐specific incompatibilities. In conclusion, we suggest that environmental conditions rather than biotic interactions likely shaped the assembly of local *Nothobranchius* communities. We acknowledge, however, that regional species pool in our study was small, limiting inferences from our analyses on the nestedness and checkerboard pattern to our particular study system.

Within clades, no sympatric co‐existence of sister species was found. *Nothobranchius furzeri* and *N. kadleci* are known to produce viable and fertile hybrids in the laboratory (Ng'oma, Groth, Ripa, Platzer, & Cellerino, 2014). It is currently assumed that allopatric speciation is the single driver of *Nothobranchius* diversification (Dorn et al., 2014). However, *Nothobranchius* genomes are evolving at a fast rate (Reichwald et al., 2015; Valenzano et al., 2015), with intra‐specific differentiation in the sex determination system in *N. furzeri* (Valenzano et al., 2015). Divergence in sex determination systems and sex‐linked genes is known to drive speciation across several taxa (Kitano et al., 2009; Qvarnström & Bailey, 2009). Interestingly, species of the R‐clade differ cytogenetically (Shidlovskiy et al., 2010), and it is possible that a combination of allopatric diversification and major cytogenetic incompatibilities during secondary sympatry may promote speciation at least in some *Nothobranchius* lineages. The parapatric distribution between *N. pienaari* and *N. rachovii*, where their populations were located within a single coastal swamp system as close as 5 km apart, is generally consistent with this scenario. If post‐mating barriers are indeed fully developed in this species pair (Shidlovskiy et al., 2010), it remains to be investigated whether a lack of premating barriers or ecological exclusion interferes with the local co‐existence of the two species. *Nothobranchius* species are certainly a promising system in which to study prezygotic, postzygotic and ecological barriers to speciation (Ng'oma et al., 2014; Polačik & Reichard, 2011; Reichard & Polačik, 2010; Sedláček, Baciaková, & Kratochvíl, 2014; Valenzano et al., 2015).

Other lineages of annual killifishes are distributed in the Neotropics. While the distribution of all annual killifishes is restricted to ephemeral pools (Polačik & Podrabsky, 2015), such pools vary in their connectivity with permanent water systems. South American annual fishes co‐exist with a diverse community of non‐annual fishes (Lanés et al., 2016; Loureiro et al., 2015; Nico & Thomerson, 1989). Unlike in the African annual fishes, particularly good understanding of community assembly exists for communities of annual *Austrolebias* fishes in Uruguay (García et al., 2009; Loureiro et al., 2015). Neotropical *Austrolebias* species are of a comparable size to the African *Nothobranchius* and co‐exist in communities comprising up to five species (Laufer et al., 2009; Loureiro et al., 2015). The distribution of individual species is best explained by past allopatric fragmentations and subsequent range expansion involving secondary contacts (García et al., 2009), likely driven by recent (Holocene) repeated marine transgressions (Sprechman, 1978). Stable species co‐existence was suggested to be mediated by inter‐specific differences in developmental time (Laufer et al., 2009), morphological specializations to diversified feeding niches (Costa, 2009; Keppeler et al., 2015) and spatial segregation of preferred microhabitats (Loureiro et al., 2015). At a regional scale, significant checkerboard pattern with a strict lack of co‐occurrence between certain species pairs was detected (Loureiro et al., 2015). *Austrolebias* species inhabit pools closely related to the active stream alluvia (Loureiro et al., 2015), making it easier to disperse at least in the downstream direction. Yet, there are strict regional limits to species occurrences (Loureiro et al., 2015; Volcan, Gonçalves, Lanés, & Guadagnin, 2015), suggesting that competitive interactions restrict potential range expansions and co‐existence of related *Austrolebias* species. The ranges of individual species vary from extensive to locally endemic (Loureiro et al., 2015; Volcan et al., 2015), being analogous to the range size variation seen in *Nothobranchius* (Reichard, 2015). Overall, historical and local effects interact to shape *Austrolebias* community assembly and abrupt limits of species distributions in a similar way to the African *Nothobranchius* species.

In conclusion, we found large co‐occurrence of all three clades across Southern and Central Mozambique, with F‐clade being absent in the region north of the Zambezi River. We demonstrated negative and positive co‐occurrence of particular *Nothobranchius* species, including a checkerboard pattern with the lack of exclusive co‐existence of the F‐ and R‐clades species in two‐species communities. We argue that, at the level of clades of closely related species, co‐occurrence patterns arise from specific associations with environmental characteristics rather than from biotic interactions and environmental factors associated with altitudinal and precipitation gradients apparently drive geographic distribution. The species with most widespread populations had also the highest relative abundance in local communities. Sister species never co‐existed. This suggests a major role for geographic isolation in speciation and potential for competitive exclusion in co‐existence of sister species. Given that other aspects of *Nothobranchius* ecology are relatively well known (Cellerino et al., 2016), this group of fish presents a useful model for evolutionary and ecological research.

## Authors Contributions

M.R., M.P., R.B. and M.V. collected field data. M.J. and M.R. analyzed data. M.R. conceived the project and drafted the manuscript. All authors contributed to the final text.

## Conflict of Interest

None declared.
